# Phytic Acid Inhibits Lipid Peroxidation *In Vitro*

**DOI:** 10.1155/2013/147307

**Published:** 2013-10-24

**Authors:** Alicja Zajdel, Adam Wilczok, Ludmiła Węglarz, Zofia Dzierżewicz

**Affiliations:** ^1^Department of Biopharmacy, Medical University of Silesia, 40-055 Katowice, Poland; ^2^Department of Biochemistry, Medical University of Silesia, 40-055 Katowice, Poland

## Abstract

Phytic acid (PA) has been recognized as a potent antioxidant and inhibitor of iron-catalyzed hydroxyl radical formation under *in vitro* and *in vivo* conditions. Therefore, the aim of the present study was to investigate, with the use of HPLC/MS/MS, whether PA is capable of inhibiting linoleic acid autoxidation and Fe(II)/ascorbate-induced peroxidation, as well as Fe(II)/ascorbate-induced lipid peroxidation in human colonic epithelial cells. PA at 100 *μ*M and 500 *μ*M effectively inhibited the decay of linoleic acid, both in the absence and presence of Fe(II)/ascorbate. The observed inhibitory effect of PA on Fe(II)/ascorbate-induced lipid peroxidation was lower (10–20%) compared to that of autoxidation. PA did not change linoleic acid hydroperoxides concentration levels after 24 hours of Fe(II)/ascorbate-induced peroxidation. In the absence of Fe(II)/ascorbate, PA at 100 *μ*M and 500 *μ*M significantly suppressed decomposition of linoleic acid hydroperoxides. Moreover, PA at the tested nontoxic concentrations (100 *μ*M and 500 *μ*M) significantly decreased 4-hydroxyalkenal levels in Caco-2 cells which structurally and functionally resemble the small intestinal epithelium. It is concluded that PA inhibits linoleic acid oxidation and reduces the formation of 4-hydroxyalkenals. Acting as an antioxidant it may help to prevent intestinal diseases induced by oxygen radicals and lipid peroxidation products.

## 1. Introduction

Free radicals in living organisms play an important role by their beneficial and harmful effects in both physiological and pathological processes. Excessive generation of free radicals can induce lipid peroxidation and oxidative damage of other biomolecules. Lipid peroxidation leads to the formation of a number of different chain length saturated and unsaturated aldehydes and other carbonyl compounds, which contribute to peroxidative cell damage by inhibiting DNA, RNA, and protein synthesis, blocking respiration and depleting glutathione pool (see [1] for review). These compounds, especially 4-hydroxyalkenals, are sufficiently long-lived products to attack target molecules distant from the site of formation and to impair their structure/function. The two most toxic 4-hydroxyalkenals are 4-hydroxynonenal (HNE) and 4-hydroxyhexanal (HHE) [2, 3]. Uncontrolled lipid oxidation has been shown to be involved in the development of many different pathological conditions such as age-related diseases, malignancy, infective diseases, and injuries [1–3]. However, these diseases may have multifactorial origin including oxidative stress derivation. Furthermore, several environmental risk factors may influence—usually accelerate or enhance—the harmful effects of free radicals. Oxidative stress results from an imbalance between free radical formation and their elimination, so antioxidants are very important players in the battle against excessive free radical generation. Therefore, there is still an increasing interest in developing efficient antioxidants that can protect against cell injury without showing toxic effects [4, 5].

Phytic acid (PA), also known as inositol hexaphosphate, inositol hexakisphosphate, IP6, or InsP6, is the main storage form of phosphorus in plants and is especially abundant in grains, nuts, legumes, and oil seeds, where it can make up 1%–5% of the edible portion. This compound demonstrates various biological activities such as antioxidant [6], anticarcinogenic [7–9], and hypoglycemic or hypolipidemic [10, 11]. The lower inositol phosphates, such as IP4 and IP3, may play roles in mediating cellular responses and have been noted as having a function in second messenger transduction systems. Dietary PA possesses ability to bind minerals, toxic trace elements, proteins and, thus, to influence their solubility, absorption, and digestibility. PA complexes with these elements are insoluble at physiological pH, and consequently, they exhibit low bioavailability. For several years PA had been considered as an antinutrient, but recent papers have shown the opposite [9–12]. Regarding its relatively high binding affinity towards minerals, especially to iron, PA has been recognized as a potent antioxidant and inhibitor of iron-catalyzed hydroxyl radical formation via the Fenton-type reaction [6, 12, 13]. Although the antioxidant capacity of PA has been documented in food processing, there is a lack of data on its effect on the formation of hydroperoxides as primary products and aldehydes as secondary products of lipid peroxidation.

The aim of the present study was to investigate whether PA is capable of inhibiting linoleic acid autoxidation and Fe(II)/ascorbate-induced peroxidation, as well as Fe(II)/ascorbate-induced lipid peroxidation in cultured Caco-2 cells. The Caco-2 cells derived from a human colon adenocarcinoma are commonly used as *in vitro* model because they spontaneously differentiate to form confluent monolayer of polarized cells structurally and functionally resembling the small intestinal epithelium. To avoid data misinterpretation resulting from the use of nonspecific analytical methods, the amounts of particular lipid hydroperoxides and hydroxyalkenals formed during lipid peroxidation in the presence of PA were measured by HPLC/DAD and HPLC/MS/MS.

## 2. Material and Methods

### 2.1. DPPH Radical Scavenging Activity

PA (phytic acid sodium salt hydrate from rice) was obtained from Sigma Chemical Co. Free radical scavenging activity of PA was determined with the use of 1,1-diphenyl-2-picrylhydrazil (DPPH; Sigma Chemical Co.) following the methodology described by Gülçin et al. [14], wherein the bleaching rate of a stable free radical DPPH was monitored at 517 nm. In its radical form, DPPH absorbs at 517 nm, but upon reduction by an antioxidant or a radical species its absorption decreases. Briefly, 0.1 mM solution of DPPH in ethanol was prepared and 1 mL of this solution was added to 3 mL of PA solution in water at different concentrations (1–500 *μ*M). After 30 minutes, the absorbance was measured at 517 nm. The capability of scavenging the DPPH radical was calculated using the following equation:

(1) $$\begin{array}{c} \text{DPPH}\;\text{radical}\;\text{scavenging}\;\text{effect}\;\left(\%\right)=\left[\frac{\left(A_{0}-A_{1}\right)}{A_{0}\;}\right]\times 100, \end{array}$$

where *A*_0_ is the absorbance of the control reaction and *A*_1_ is the absorbance measured in the presence of PA.

### 2.2. Ferrous Ions Chelating Activity

The chelation of ferrous ions by PA was estimated by the method of Dinis et al. [15]. PA at various concentrations (1–500 *μ*M) in water (0.4 mL) was added to the solution of 2 mM FeCl_2_ (0.05 mL). The reaction was initiated by the addition of 5 mM ferrozine (0.2 mL, Sigma Chemical Co.), and the total volume was adjusted to 4 mL with ethanol. The mixture was shaken vigorously and left at room temperature for 10 minutes. Absorbance of the solution was measured spectrophotometrically at 562 nm. The percentage of inhibition of ferrozine-Fe(II) complex formation was calculated using the following formula:

(2) $$\begin{array}{c} \text{Metal}\;\text{chelating}\;\text{effect}\;\left(\%\right)=\left[\frac{\left(A_{0}-A_{1}\right)}{A_{0}}\right]\times 100, \end{array}$$

where *A*_0_ is the absorbance of control and *A*_1_ is the absorbance measured in the presence of PA. The control contained FeCl_2_ and ferrozine.

### 2.3. Measurement of Linoleic Acid Oxidation 

#### 2.3.1. Linoleic Acid Oxidation

Linoleic acid (Sigma Chemical Co.) micelles were used as model lipid system to determine the effect of PA on autoxidation or induced lipid peroxidation. The reaction mixtures contained linoleic acid (1 mM) and dispersed in HEPES buffer (50 mM, pH 7.4; Sigma Chemical Co.) and PA at various concentrations (1–500 *μ*M). Linoleic acid peroxidation was induced by adding ferrous ions (FeCl_2_, Sigma Chemical Co.) and ascorbic acid (Sigma Chemical Co.) to the final concentrations of 20 *μ*M and 100 *μ*M, respectively. Incubations were carried out for 24 hours at 37°C in a water bath with gently shaking. After incubation, each sample was diluted with methanol (1 : 1; vol/vol), filtered (filter Millex GV13, pore diameter 0.22 *μ*m; Millipore), and immediately analyzed by HPLC. 

#### 2.3.2. HPLC Analysis of Linoleic Acid and Its Hydroperoxides

Analytical reverse-phase HPLC was performed with a Hewlett-Packard model 1050 liquid chromatograph equipped with a HP 1100 diode array detector and interfaced to HPLC ChemStation A.06.03 (HP). The incubation products were separated on the Eurospher 100 C18 column (particle size 5 *μ*m, 250 × 4 mm; Saulentechnik Knauer) eluted isocratically with acetonitrile : water : phosphoric acid (80 : 20 : 0.1) at a flow rate of 1 mL/min at 35°C. The eluent was monitored at 234 nm for linoleic acid hydroperoxides formation and at 206 nm for linoleic acid disappearance. These compounds were identified by matching their retention times and UV spectra with authentic standards. Quantification was made based on the calibration curves. 

### 2.4. Measurement of Secondary Products of Lipid Peroxidation in Caco-2 Cells

#### 2.4.1. Cell Culture

Caco-2 cells were purchased from the German Collection of Microorganisms and Cell Cultures (Braunschweig, Germany). The cells were grown in RPMI 1640 medium (Sigma Chemical Co.) supplemented with 10% fetal bovine serum (FBS; Gibco BRL), 100 U/mL penicillin, 100 *μ*g/mL streptomycin, and 10 mM HEPES (Gibco). The cell cultures were maintained at 37°C in a 5% CO_2_ atmosphere.

PA at nontoxic concentrations (0.1 mM or 0.5 mM) [16] was added to the cell cultures 1 hour prior the induction of peroxidation by Fe(II) and ascorbic acid (20 *μ*M and 100 *μ*M, resp.). Control cells were treated with Fe(II)/ascorbic acid without PA addition and cultured for 24 hours.

#### 2.4.2. Preparation of the Dinitrophenylhydrazone (DNP) Derivatives of 4-Hydroxyalkenals

DNP derivatives of the standard aldehydes were prepared according to the commonly used method described by Esterbauer et al. [17]. After 24 hours in culture, the cells were washed twice with PBS, harvested by scraping, centrifuged (10 000 g, 10 min), and immediately frozen in liquid nitrogen. The frozen cells (40–60 mg) were mixed with 1 mL of methanol, vortexed (10 min) for extraction, and again centrifuged (10 000 g, 10 min). The extraction was repeated twice. The supernatants were combined and added to the equal volumes of the DNPH reagent. The derivatization was carried out for 1 hour in dark at room temperature. The DNPH reagent was freshly prepared by dissolving 35 mg of 2,4-dinitrophenylhydrazine (Sigma Chemical Co.) in 100 mL of 1 M HCl and extracted twice with 50 mL hexane for removal of impurities. The obtained derivatives after a subsequent centrifugation (10 000 g, 15 min) were dissolved in 0.3 mL of acetonitrile and filtered (0.22 *μ*m, Millipore). This procedure was adapted from a similar method proposed for malondialdehyde (MDA) and HNE derivatization by Deighton et al. [18].

#### 2.4.3. Liquid Chromatography/Mass Spectrometry Determination

The 20 *μ*L samples containing DNP-derivatives were injected on a C-18 column (Saulentechnik Knauer; 250 × 2 mm, 5 *μ*m) and separated using a gradient mixture of water, methanol, and acetonitrile acidified with formic acid on the HPLC system HP 1100 with a diode array detector from which the effluent was directed to the API 2000 PE-Sciex mass spectrometer. The following gradient program was used for elution: 0 min – methanol : acetonitrile : water : formic acid (20 : 30 : 50 : 0.1), 10 min – methanol : acetonitrile : water : formic acid (20 : 60 : 20 : 0.1), 14 min – methanol : acetonitrile : water : formic acid (20 : 80 : 0 : 0.1). The other chromatographic parameters were as follows: flow rate 0.4 mL/min, temperature 35°C, run time 35 min, post time 12 min, and DAD detection range 200–400 nm.

The mass spectrometer equipped with atmospheric pressure ionization source worked in the negative ionization mode. The ion source and detector parameters were identical in each run (CUR 50; IS −3800 V; TEM 350; GS1 60; GS2 85; CAD 2; CE 18 eV; DP −25; FP −200; EP 10). Detection was performed in multiple reaction monitoring (MRM) mode, using characteristic mass pairs specific for each analyzed compound (*m/z* 293→167 for 4-hydroxyhexanal, *m/z* 335→167 for 4-hydroxynonenal). The concentrations of HHE and HNE were related to the amount of total cell protein determined by the standard Bradford method.

### 2.5. Statistical Analysis

The data obtained from 3 independent series of experiments (each in triplicate) were expressed as mean values ± standard deviations. Statistical significance analysis was based on analysis of variance (ANOVA) followed by Tukey's HSD test. The *P* value of less than 0.05 was considered significant. Statistical analysis was performed using Statistica 10 PL software for Windows (StatSoft, Poland).

## 3. Results and Discussion 

A number of methods have been developed to determine the antioxidant capacities of chemical compounds and to evaluate different antioxidant mechanisms [19]. The study presented in this paper has focused on the measurement of inhibition of linoleic acid autoxidation and catalytic oxidation and Fe(II)/ascorbate-induced lipid peroxidation in a human colon Caco-2 cells by PA with the use of the HPLC/MS/MS method.

In the present study, the antioxidant properties of PA at its various concentrations (1–500 *μ*M) were examined by using the method based on DPPH scavenging and ferrous ions chelating ability determination. PA in the above concentration range did not scavenge DPPH radical (*P* > 0.05). These results are in agreement with earlier findings, where the scavenging effect of PA was observed after its irradiation only and it was positively correlated with irradiation dose [20, 21]. Ahn et al. [22] conducted a similar study to evaluate antioxidant activities of irradiated PA and ascorbic acid. Irradiated PA showed a significantly higher DPPH radical scavenging activity than ascorbic acid at the same concentration (800 *μ*M). 

**Figure 1:**
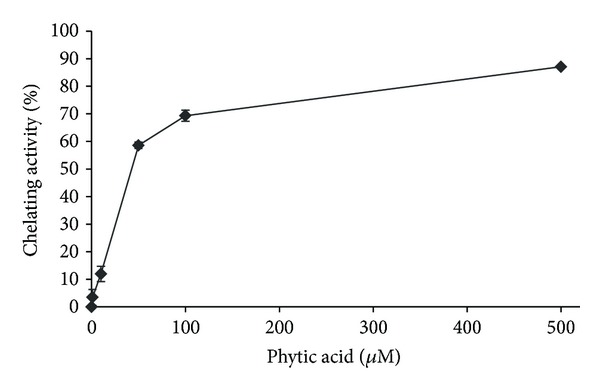
Ferrous ions chelating effect of phytic acid at various concentrations (0–500 *μ*M) measured by ferrozine/FeCl_2_. Results are the mean ± SD of 3 experiments.

One of the most commonly used systems for testing the chelating activity on Fe(II) is ferrozine/FeCl_2_. The PA chelating activity in this system is presented in Figure 1. The observed effects were concentration dependent. PA exhibited 11.9, 58.6, 69.3, and 87.1% of ferrous ions chelation at 10, 50, 100, and 500 *μ*M, respectively. These results confirm the findings that PA is an antioxidant acting as a potent inhibitor of iron-catalyzed radical formation by chelating free iron and blocking its coordination sites. Sakač et al. [23] observed the inhibition of hydroxyl radical generation over a wide range of phytate/iron ratio (1 : 5.5–1 : 22) and found that one phytate molecule could bind up to 6 divalent cations. At the concentration of 117 *μ*M, PA exhibited chelating activity of 96% in ferrozine/FeSO_4_ system [23]. It was demonstrated with the use of BPS (bathophenanthroline) based method that both standard PA and purified PA obtained from rice bran, exhibited an iron chelating capacity, which was dependent on its concentration and contact time with iron before adding BPS [24]. Ahn et al. [22] confirmed the antioxidant activity of PA by the ferric reducing antioxidant power (FRAP) test.

**Figure 2:**
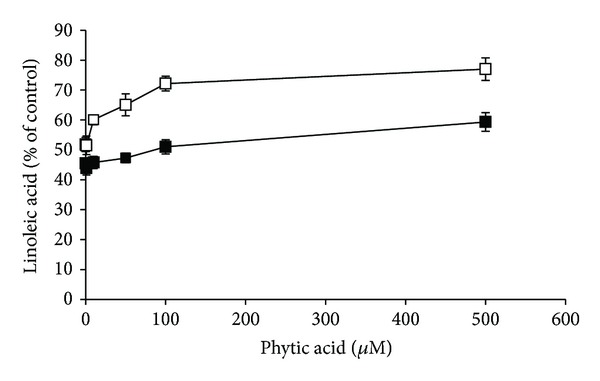
Effect of phytic acid (0–500 *μ*M) on the 24-hour lasting autoxidation (□) and Fe(II)/ascorbate-induced peroxidation (■) of linoleic acid expressed as percent of the control. Results are the mean ± SD of 5 experiments.

**Figure 3:**
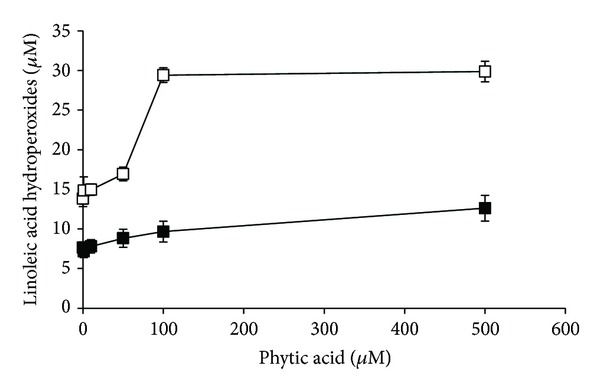
Effect of phytic acid (0–500 *μ*M) on linoleic acid hydroperoxide concentration (*μ*M) after 24-hour lasting autoxidation (□) and Fe(II)/ascorbate induced linoleic acid peroxidation (■). Results are the mean ± SD of 5 experiments.

Transition metal ions are involved in lipid peroxidation by decomposing lipid peroxides into their peroxyl and alkoxyl radicals. These radicals abstract hydrogen atom from polyunsaturated fatty acid molecule and perpetuate a chain reaction of lipid peroxidation [1]. PA has antioxidant functions by virtue of forming a unique iron chelate, and it suppresses iron-catalyzed oxidative reactions [6]. Consequently, such actions of PA may be involved in its inhibitory effect on lipid oxidation [13]. Therefore, in the present study it was investigated whether PA was able to inhibit autoxidation and Fe(II)/ascorbate-induced peroxidation of linoleic acid. In this analysis, the oxidant Fe(II)/ascorbate pair was used because iron can be reduced by ascorbate and, thus, cause a significant increase in the formation of reactive oxygen species and the extent of lipid peroxidation [25]. As shown in Figure 2, PA at 100 *μ*M and 500 *μ*M effectively inhibited the decay of linoleic acid, both in the absence and presence of Fe(II)/ascorbate (*P* < 0.05). The observed inhibitory effect of PA on Fe(II)/ascorbate-induced lipid peroxidation was lower (10–20%) compared to that of autoxidation, probably due to its direct interaction with Fe(II) ions. PA did not change linoleic acid hydroperoxides concentration levels after 24 hours of Fe(II)/ascorbate-induced peroxidation (*P* > 0.05), and only about 3% of linoleic acid was converted into hydroperoxides (Figure 3). In the absence of Fe(II)/ascorbate, PA at 100 *μ*M and 500 *μ*M significantly suppressed decomposition of linoleic acid hydroperoxides (*P* < 0.05; Figure 3). Their concentrations increased about twofold. With the use of 100 *μ*M and 500 *μ*M PA, about 10% and 13% of linoleic acid were converted into hydroperoxides. These results are in agreement with the findings of Graf et al. [13], who demonstrated that phytate prevented peroxidation of arachidonic acid driven by ascorbic acid and iron by shifting the redox potential of iron Fe(II) → Fe(III). Fe(II) stimulates production of lipid oxyradicals, whereas Fe(III) is relatively inert. Studies using liposomal membranes demonstrated that PA derived hydrolytic products containing three or more phosphate groups were able to inhibit iron induced lipid peroxidation, although their effectiveness decreased with dephosphorylation degree. The products of PA hydrolysis also prevented iron induced decomposition of phosphatidylcholine hydroperoxide [26]. Ahn et al. [20] reported that the antioxidant activity of PA was slightly increased by irradiation in an aqueous lipid model system; although at higher concentrations (400 *μ*g/mL), this activity remained the same compared to that of nonirradiated PA or it was even reduced.

Antioxidants may inhibit decomposition of lipid hydroperoxides by acting as radical scavengers, metal chelators, or reducers of hydroperoxides to more stable hydroxyl compounds [1]. These effects of PA are mainly associated with its chelating activity. The results of the present study suggest that PA at higher concentration (100 *μ*M, 500 *μ*M) scavenged the reactive oxygen species produced during autoxidation of linoleic acid and prevented decomposition of lipid hydroperoxides. On the other hand, the earlier report postulated, that PA did not act as the chain-breaking antioxidant capable of scavenging free radicals and thus preventing lipid oxidation. This can be explained by the structural feature of PA which is a lack of a hydrogen atom to be transferred to peroxyl radicals [22, 23]. This conclusion was later confirmed by the results of AOA (*β*-carotene bleaching) test in *β*-carotene/linoleic acid model system. PA did not exhibit any antioxidant activity in the tested concentration range (3.7–58.6 *μ*M) [23]. Sakač et al. [23] found that it did not inhibit thermal oxidation of hydroperoxide-enriched soybean oil (HESO) but influenced catalytic oxidation of HESO by chelating Fe(II) and inhibited generation of lipid oxyradicals that were detected in the form of PBN-OOL/-OL spin adducts.

The possibility of utilizing PA as an antioxidant in food processing and storage was documented by Stodolak et al. [27], who found the improvement of oxidative stability of raw and cooked meat. It is known that PA can effectively and dose-dependently inhibit lipid peroxidation in beef and pork homogenates [28, 29]. Park et al. [21] concluded that irradiated PA significantly inhibited lipid oxidation in meat homogenates compared to the control sample. Moreover, it was shown that irradiated PA was capable of preventing the loss of the heme iron and myoglobin formation during meat storage. Sorour and Ohshima [30] confirmed the antioxidant potential of pure PA (PA sodium salt) and PA extracted from wheat bran. They measured TBARS level, oxygen absorption, and total lipid hydroperoxide content in cod liver oil o/w emulsion oxidized at 40°C under dark. PA at a concentration of 4 mM inhibited total hydroperoxides and TBARS formation by 62% and 67% of control, respectively. In this study, PA appeared to be more effective than ascorbic acid and therefore it was recommended as a food antioxidant that prolongs the stability of fish lipids or fish meats [30]. 

Lipid peroxidation has been described to cause gradual changes in cellular membrane structure, ultimately leading to the loss of function and integrity [1]. Moreover, aldehydic lipid peroxidation products, especially 4-hydroxyalkenals (HHE, HNE), due to their high reactivity, show marked biological effects. Experimental and clinical evidence suggest that 4-hydroxyalkenals can act as bioactive molecules under physiological and/or pathological conditions. These compounds can affect and modulate, even at very low-nontoxic concentrations, several cell functions, including signal transduction, gene expression, cell proliferation, and differentiation, cellular growth inhibition, or apoptosis [2, 3]. Numerous methods have been developed to measure lipid peroxidation products and lipid peroxidation damage in tissues, cells, and body fluids. Malondialdehyde (MDA) reactivity with 2-thiobarbituric acid (TBA) is the principal analytical method for evaluating lipid peroxidation [31]. Nevertheless, the specificity of these methods can be questioned because aldehydes other than MDA can react with TBA. Moreover, TBA assay conditions such as high temperature and low pH may themselves cause the oxidation of lipids. Therefore, several analytical methods have been proposed using chromatographic techniques coupled with sensitive detectors for determination of aldehydic products in biological systems [32, 33]. In the present study, the HPLC/MS/MS method was applied for the quantitative analysis of aldehydic lipid peroxidation products formed in Caco-2 cells. PA at the tested nontoxic concentrations (100 *μ*M and 500 *μ*M) significantly decreased HHE and HNE levels (about twofold) in Caco-2 cells (Table 1). The obtained results correspond with the findings that dietary intrinsic phytate from corn and soy was protective against lipid peroxidation in the colon of pigs [34], rats [35], and mice [36] supplemented with a moderately high level of dietary iron. Another experiment performed on high fat-fed mice receiving rice bran or pure PA in the diet showed that such supplementation suppressed lipid peroxidation as evidenced by the significantly lower TBARS levels in plasma and erythrocytes [10]. Biological activity of PA *in vivo* in most cases seems to be associated with its antioxidant activity, chelation of Fe(III), and suppression of hydroxyl radical formation. Although this mechanism is widely recognized data on antioxidant effect of PA *in vivo* published in the recent years are very limited. 

## 4. Conclusion

PA can scavenge the reactive oxygen species produced during autoxidation of linoleic acid and reduce the formation of 4-hydroxyalkenals. It can act as a natural antioxidant and prevent intestinal diseases induced by oxygen radicals and lipid peroxidation products. 

## Figures and Tables

**Table 1:** Effect of phytic acid on aldehydic lipid peroxidation product levels in Caco-2 cells after 24-hour lasting Fe(II)/ascorbate-induced lipid peroxidation (mean ± SD of 4 experiments).

Phytic acid [mM]	4-Hydroxyhexenal [nmol/g protein]	4-Hydroxynonenal [nmol/g protein]
0	8.24 ± 1.69	0.44 ± 0.09
0.1	3.89 ± 0.93*	0.24 ± 0.07*
0.5	2.83 ± 0.89*	0.18 ± 0.06*

*Statistically significant difference in comparison with control; *P* < 0.05.
